# Intergenerational transmission of genetic risk for hyperactivity and inattention. Direct genetic transmission or genetic nurture?

**DOI:** 10.1002/jcv2.12222

**Published:** 2024-03-04

**Authors:** Ivan Voronin, Isabelle Ouellet‐Morin, Amélie Petitclerc, Geneviève Morneau‐Vaillancourt, Mara Brendgen, Ginette Dione, Frank Vitaro, Michel Boivin

**Affiliations:** ^1^ École de psychologie Université Laval Québec Quebec Canada; ^2^ School of Criminology University of Montreal The Research Center of the Montreal Mental Health University Institute and the Research Group on Child Maladjustment Montréal Quebec Canada; ^3^ Social, Genetic & Developmental Psychiatry Centre Institute of Psychiatry, Psychology & Neuroscience King's College London London UK; ^4^ Département de Psychologie Université du Québec à Montréal Montréal Quebec Canada; ^5^ École de Psychoéducation Université de Montréal Montréal Quebec Canada

**Keywords:** ADHD, educational attainment, hyperactivity, inattention, intergenerational transmission, polygenic score

## Abstract

**Background:**

Hyperactivity and inattention, the symptoms of ADHD, are marked by high levels of heritability and intergenerational transmission. Two distinct pathways of genetic intergenerational transmission are distinguished: direct genetic transmission when parental genetic variants are passed to the child's genome and genetic nurture when the parental genetic background contributes to the child's outcomes through rearing environment. This study assessed genetic contributions to hyperactivity and inattention in childhood through these transmission pathways.

**Methods:**

The sample included 415 families from the Quebec Newborn Twin Study. Twins' hyperactivity and inattention were assessed in early childhood by parents and in primary school by teachers. The polygenic scores for ADHD (ADHD‐PGS) and educational attainment (EA‐PGS) were computed from twins' and parents' genotypes. A model of intergenerational transmission was developed to estimate (1) the contributions of parents' and children's PGS to the twins' ADHD symptoms and (2) whether these variances were explained by genetic transmission and/or genetic nurture.

**Results:**

ADHD‐PGS explained up to 1.6% of the variance of hyperactivity and inattention in early childhood and primary school. EA‐PGS predicted ADHD symptoms at both ages, explaining up to 1.6% of the variance in early childhood and up to 5.5% in primary school. Genetic transmission was the only significant transmission pathway of both PGS. The genetic nurture channeled through EA‐PGS explained up to 3.2% of the variance of inattention in primary school but this association was non‐significant.

**Conclusions:**

Genetic propensities to ADHD and education predicted ADHD symptoms in childhood, especially in primary school. Its intergenerational transmission was driven primarily by genetic variants passed to the child, rather than by environmentally mediated parental genetic effects. The model developed in this study can be leveraged in future research to investigate genetic transmission and genetic nurture while accounting for parental assortative mating.

## Graphical abstract

We proposed a novel model to study intergenerational transmission of genetic risk using the polygenic score data from twin families. The model revealed that the polygenic scores for ADHD and educational attainment were contributed to individual differences in hyperactivity and inattention in childhood, with an increasing contribution in primary school. The intergenerational transmission of genetic risk occurred primarily through genetic variants immediately passed on from parents to children.

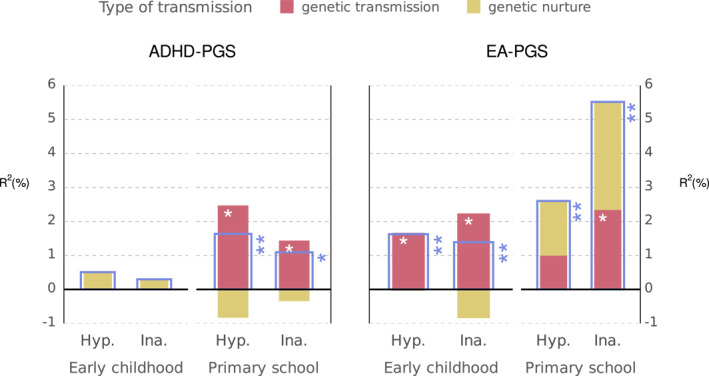

**Key points**

- Recent research shows two pathways of genetic intergenerational transmission: through (1) genetic variants transmitted from parent to child and (2) heritable parental behaviors that influence the child's developmental context.
- Genetic propensities toward ADHD and education both contributed to individual differences in hyperactivity and inattention in childhood, with an increase in contributions noted during primary school.
- Genetic propensity toward education was shown to be a more powerful predictor of ADHD problems than a genetic propensity toward ADHD.
- Direct genetic transmission was the primary mechanism of intergenerational transmission of ADHD.
- We propose a model of intergenerational transmission within a structural equation modeling framework that can be leveraged in future research.

## INTRODUCTION

Attention‐deficit/hyperactivity disorder (ADHD) is an early‐onset neurodevelopmental condition characterized by age‐inappropriate and persistent levels of inattention, impulsivity and hyperactivity (Franke et al., 2018). About 5%–10% of children and adolescents worldwide typically meet the diagnostic criteria for ADHD, with a higher prevalence among boys (Ayano et al., 2020; Polanczyk et al., 2014; Wang et al., 2017). Hyperactivity and inattention (ADHD) symptoms predict a variety of adjustment difficulties, including learning problems (Mayes et al., 2000; Reale et al., 2017), depression and anxiety (Eyre et al., 2019; Reale et al., 2017), sleep disturbances (Miano et al., 2019), antisocial and substance use problems (Retz et al., 2021). Hyperactivity and inattention are highly heritable, with 50%–70% of interindividual differences explained by genetic factors in twin studies (Faraone & Larsson, 2019; Pingault et al., 2015; Plourde et al., 2015). Genes accounting for ADHD symptoms in the general population largely overlap with those in clinical populations (H. Larsson et al., 2012; Stergiakouli et al., 2015), and this genetic risk is massively polygenic (Demontis et al., 2019, 2023). The early genetic underpinnings of ADHD are largely responsible for their phenotypic stability, although new emerging contributions in childhood have also been detected (Kuntsi et al., 2005; J.‐O. Larsson et al., 2004). While, hyperactivity and inattention share a part of their genetic basis (Greven, Rijsdijk, et al., 2011; Nikolas & Burt, 2010), there is a substantial genetic specificity that links inattention to cognitive deficit, learning disability and educational outcomes (DuPaul & Volpe, 2009; Greven, Harlaar, et al., 2011; Kuntsi et al., 2014), and hyperactivity to oppositional and substance use problems (Quinn et al., 2016; Wood et al., 2009).

The substantial role of genetics in ADHD symptoms, and the parent‐child correlation in ADHD symptoms (Uchida et al., 2021) warrant the research into their intergenerational transmission. Each biological parent transmits half of their genetic variants to the child via genetic or Mendelian inheritance. In addition to this direct transmission, parents also provide a developmental context possibly influenced by their own genetics, including their non‐transmitted genetic propensities, which can foster the child's ADHD symptoms. For example, maternal smoking, alcohol consumption and substance use are all putative environmental risks for children's ADHD (Knopik et al., 2019). Children with ADHD are more likely to grow up in poor families (Hill et al., 2015) and to experience family stress (e.g., parent‐child conflicts) (Deault, 2010; Harold et al., 2013; Sellers et al., 2021). Crucially, parental ADHD symptoms predict child's ADHD symptoms and comorbidities (Agha et al., 2013; Vizzini et al., 2019), and many prenatal and familial risks of the latter are also partly associated with genetic differences (Boivin et al., 2005; Hyytinen et al., 2019).

Thus, parental genetic risk could foster a child's ADHD symptoms through other means than direct genetic transmission. The intergenerational transmission of genetic risk for ADHD symptoms may indeed arise from two distinct pathways (Hart et al., 2021; Koellinger & Harden, 2018). First, direct **genetic transmission** describes the transmission of genetic risk through DNA. Second, **genetic nurture** refers to the putative contribution of parents' genetic risk to their child's trait beyond direct transmission, including through parenting, parent‐child interactions, and environments they create for their child.

Research designs can now use polygenic scores (PGS) to disentangle these two pathways in the study of intergenerational transmission. PGS represents an individual's genetic propensity for a specific trait, which is calculated as a sum of alleles associated with a given phenotype weighted by the size of their observed association with this phenotype in so‐called discovery GWAS (Choi et al., 2020; Zheutlin & Ross, 2018). For example, a recent PGS for ADHD (ADHD‐PGS) explained 3.6%–4.0% of the variance of broadly defined ADHD (Li & He, 2021), whereas a PGS for educational attainment (EA‐PGS) accounted for 12%–16% in educational attainment (Okbay et al., 2022). A variety of research designs with PGS were proposed to estimate genetic transmission and genetic nurture. A *first* approach disentangles within‐family and between‐family variance in children's PGS, with the former reflecting genetic transmission and the difference across within‐ and between‐family contributions signaling genetic nurture (Selzam et al., 2019). Complementary to the first approach, a *second* strategy used parent and offspring genotype data to calculate two PGS: a transmitted (child's own) and a non‐transmitted PGS (computed from non‐transmitted variants). The association between the non‐transmitted PGS and the child's trait reflects genetic nurture because it can only operate through environmental mediation (Balbona et al., 2022; Bates et al., 2018; de Zeeuw et al., 2020; Kong et al., 2018). For example, Balbona et al. (2021) designed a structural equation model with PGS (SEM‐PGS) where the paths of intergenerational transmission were estimated in family trios using transmitted and non‐transmitted parental PGS to predict a measured child's trait. Another study tested direct genetic transmission by assessing whether the genetic liability to ADHD and its comorbidities, in the form of PGS, was elevated in children with ADHD, compared to the control population (Martin et al., 2023). Similarly, the genetic nurture was estimated as the difference of the non‐transmitted PGS between proband and control families. The studies with non‐transmitted polygenic scores generally show that the genetic risk for hyperactivity and inattention is passed on through direct genetic transmission rather than genetic nurture (de Zeeuw et al., 2020; Martin et al., 2023).

A *third* approach addressed the genetic nurture transmission by estimating the unique contributions of the full mother's, father's and child's PGS to the child's phenotype using regression (Pingault et al., 2022) or SEM (Axelrud et al., 2023; Frach et al., 2023). Under the former approach, a significant unique contribution of parents PGS is suggestive of genetic nurture, whereas an attenuation of parental PGS contribution when adjusting for the child's PGS indicates genetic transmission. For example, Pingault et al. (2022) used a variety of PGS, including for ADHD, as predictors of mother‐rated child's ADHD symptoms in a large cohort from Norway and found that the contribution of parent's PGS to the child's ADHD indeed decreased after controlling for the child's PGS, suggesting genetic transmission. The only unique parent's PGS contribution was detected for mother PGS for neuroticism. Frach et al. (2023) estimated the simultaneous PGS effects of parents and the child within a SEM model to show that multiple polygenic risks and propensities were involved in the transmission of childhood and adolescence externalizing problems, but primarily via direct genetic transmission (i.e., through the child's own genotype). Significant genetic nurture effects were detected for cognitive performance and educational attainment, but this result was not replicated. Finally, Axelrud et al. (2023) tested whether the contribution of parental PGS to child's cognition, education and psychopathology (schizophrenia and ADHD) was mediated by the child's PGS (direct genetic transmission) and/or by parental level of education and psychopathology symptoms (genetic nurture). This study found that both pathways were involved in the transmission of genetic propensity of educational and cognitive traits, however, the transmission of psychopathology effectively was not addressed because none of parental PGS significantly predicted child's psychopathology (Axelrud et al., 2023).

Thus, intergenerational transmission has been examined through a variety of designs, each with its own strengths and limitations. For instance, in addition to the limitation of missing data and thus the needed imputation, the regression design does not provide a direct estimate of the correlation between parents' PGS that may result from assortative mating and population stratification. Assortative mating refers to a non‐random mating that results in elevated phenotypic similarity of spouses/parents pertaining to various traits, such as education and psychopathology, including ADHD (Boomsma et al., 2010; Nordsletten et al., 2016). To the extent that it contributes to genetic similarity between the parents (Peyrot et al., 2016), assortative mating inflates genetic resemblance between family members (Torvik et al., 2022) and biases the estimates of genetic association and heritability in GWAS and PGS studies (Pingault et al., 2022; Plomin et al., 2016). Population stratification, defined as the patterns of genetic variation due to ancestry and migration, similarly contributes to elevated genetic similarity between spouses and within their family (Abdellaoui et al., 2013). Posing a risk for spurious genetic associations (Sohail et al., 2019), population stratification is commonly addressed by regressing genetic predictors, such as PGS, on principal components of the genetic relatedness matrix (Abdellaoui et al., 2013). This method, however, may fail to account for recent population history, leaving the possibility of bias (Zaidi & Mathieson, 2020). The genetic similarity between parents was modeled in the SEM‐PGS approach of Balbona et al. (Balbona et al., 2021, 2022) that also allowed for incomplete data using the full‐information maximum likelihood estimation (FIML). A possible shortcoming of this approach is that it relies on more complex and power‐demanding transmitted and non‐transmitted PGS. Finally, most of the current intergenerational transmission designs are limited to family trios, leaving out the data from the twin registers with their heuristic advantages (Boivin et al., 2019; Ligthart et al., 2019).

**FIGURE 1:**
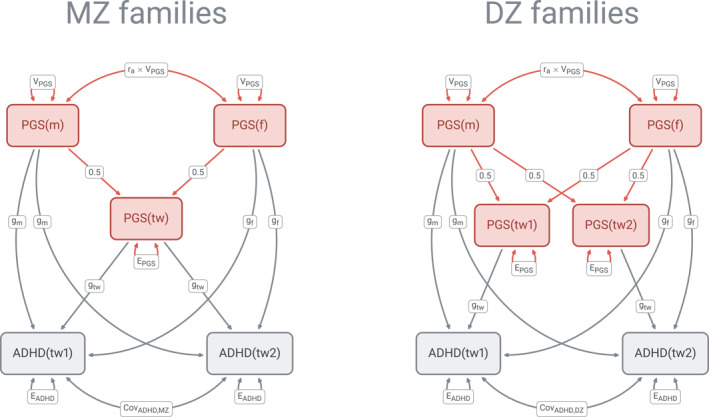
Path diagram for the intergenerational transmission model. ADHD, ADHD symptom; f, father; m, mother; PGS, polygenic score; tw1/2, twin. For parameter labels refer to Methods and Supplementary Methods.

The present study proposes an integrated model of intergenerational transmission that estimates transmission pathways from the data of twin families using the structural equation modeling (SEM) approach (Figure 1). Unique contributions of twins' and parents' PGS, as well as the parents' genetic resemblance due to assortative mating and population stratification are estimated in the model, thus enhancing precision of the estimate of each genetic transmission and genetic nurture components compared to a family trio approach. More specifically, when the same number of families are considered, the statistical power is enhanced due to the fact that twin families provide twice as much of “PGS‐phenotypic” data from which the PGS effects are estimated. We also use the estimates of the twins' and parents' PGS contributions to the child's phenotype to compute the percent of the variance of ADHD outcomes explained by the two transmission pathways, which is not commonly reported in the intergenerational research with PGS. We chose the ADHD‐PGS and the EA‐PGS as genetic predictors in our model because the ADHD‐PGS captured not only ADHD diagnosis, but population‐based distribution of hyperactivity and inattention symptoms (Demontis et al., 2019), whereas the EA‐PGS represented a broad genetic propensity predictive of school attainment and its correlates, such as socioeconomic status and disruptive behaviors, including ADHD symptoms (Jansen et al., 2018). These two PGS could thus offer complementary perspectives on the direct and indirect ADHD intergenerational genetic transmission pathways.

We aimed to address following research questions: (1) To what extent are children's ADHD symptoms predicted by genetic propensities for ADHD (ADHD‐PGS) and for educational attainment (EA‐PGS), the latter considered as a broader genetic predictor of factors, such as ADHD symptoms and especially inattention, involved in educational achievement? We expected that parents' and children's ADHD‐PGS would positively, and EA‐PGS negatively predict children's ADHD symptoms. (2) To what extent do direct genetic transmission and genetic nurture mediate the contributions of parents' PGS to their children's ADHD symptoms? We expected that the parents' ADHD‐PGS contributions would be transmitted mainly through direct genetic transmission (i.e., through the children's own genotype), whereas parents' EA‐PGS contribution would be conveyed through both direct genetic transmission and genetic nurture given EA‐PGS documented links with social capital outcomes (Belsky et al., 2016). (3) Do these patterns of transmission vary with age (early childhood vs. primary school)? We expected both genetic propensities to become more predictive of ADHD symptoms with children's increasing age. (4) Finally, do PGS prediction and transmission patterns differ for hyperactivity and inattention? We expected stronger prediction of hyperactivity symptoms through ADHD‐PGS (Quinn et al., 2016; Wood et al., 2009), whereas EA‐PGS should be more predictive of inattention (Greven, Harlaar, et al., 2011; Kuntsi et al., 2014; Pingault et al., 2014).

## METHODS

### Sample

The study sample was drawn from the Quebec Newborn Twin Study (QNTS, Boivin et al., 2019), a population‐based birth cohort of twin families from the greater Montreal area (Canada). The families were recruited when the twins were 5 months old and subsequently assessed annually or biennially on a variety of developmental outcomes and experiences. The present study involved 415 twin families (169 monozygotic and 246 dizygotic, 63% of all QNTS families) selected under the following inclusion criteria: (1) at least one available assessment of twin ADHD symptoms, (2) at least one available twin's genotype. The rest of QNTS families were excluded from the study. The resulting sample included 378 families with both twin genotypes (assuming MZ twins share identical genotypes) and 37 families with one twin genotype. Among these families, 159 had both parents' genotypes, 67 families had only one, and 189 families had no parental genotypes. Details on the available genotype data in MZ and DZ families are provided in Table S1.

The sociodemographic sample characteristics and comparison across included versus excluded QNTS families are presented in Table S2. Participating parents were mostly White (95.5%), French‐speaking (90.0%), and had 12 years of formal education on average. Most families (83.5%) included both biological parents and about half (51%) had an annual income of at least $50,000CAD. Excluded families had a lower income, a higher percentage of non‐Francophone parents, more ethnic diversity, and had children with lower levels of inattention in early childhood, but higher levels of inattention in primary school. Included and excluded families did not differ in terms of twins' hyperactivity symptoms, family status (both biological parents present in the family vs. other) or parental educational level.

### Procedures

Children's ADHD symptoms were assessed through mothers' ratings when the twins were 19, 32, 50, and 63 months old (early childhood), and through teachers' ratings when the twins were in kindergarten and in Grades 1, 3, 4, and 6 (primary school; 6, 7, 9, 10, and 12 years old, respectively). Blood or saliva samples were collected when the twins were 8 years old (407 parents and 581 twins), and at age 19 years (saliva of 328 twins, Boivin et al., 2019). DNA was extracted and genotyped at Genome Quebec, Montreal, Canada, using Illumina PsychArray‐24 v1.3 BeadChip. Quality control and imputation were performed on genotypes yielding information for 8,465,216 SNP (see details in Supporting Information S1).

### Measures

#### ADHD symptom scores

ADHD symptoms were assessed with five hyperactivity items (‘continuously agitated’, ‘impulsive, acts before thinking’, ‘can't sit still’, ‘has difficulty waiting their turn in games’, ‘can't stay calm to do something’) and three inattention items (‘get distracted’, ‘can't concentrate’, ‘is inattentive’), rated on a three‐point scale from 0 (‘never’) to 2 (‘often’) (Collet et al., 2023; Leblanc et al., 2008). Both hyperactivity and inattention items were averaged at each measurement time, and then, these resulting scores were averaged across the measurement times first, within early childhood and second, within primary school. This was justified by the high stability of these scores within each developmental period: *r*_*s*_ ≥ 0.80, *p*_*s*_ < 0.001, for early childhood hyperactivity and *r*_*s*_ ≥ 0.69, *p*_*s*_ < 0.001 for inattention; *r*_*s*_ ≥ 0.85, *p*_*s*_ < 0.001, for primary school hyperactivity and *r*_*s*_ ≥ 0.83, *p*_*s*_ < 0.001 for inattention. Each score was then square‐root transformed given non‐normality of the data. The transformation was applied within a linear model that also adjusted for sex differences in symptoms.

#### Polygenic scores

The PGS were computed in two steps. First, the summary statistics from the GWASs for ADHD (Demontis et al., 2019) and educational attainment (Okbay et al., 2022) were adjusted with a Bayesian approach implemented in the PRS‐CS software (Ge et al., 2019), and then used to compute both ADHD and EA PGSs (details in Supporting Information S1). For all statistical analyses, the estimated contributions of PGS were adjusted for population stratification using factor scores on the first 10 principal components of the genetic relatedness matrix.

### Statistical analyses

Our model of intergenerational transmission considered twins' and their parents' PGS as predictors of twins' ADHD symptoms (Figure 1) in a two‐group (MZ and DZ families) structural equation model (SEM, Loehlin, 2004). Ten parameters were estimated: the variance of PGS (*V*_PGS_), the correlation between parents' PGS (*r*_*a*_), the residual variance of the ADHD scores (*V*_ADHD_), the residual correlations between ADHD scores within MZ and DZ pairs (*r*_MZ_, *r*_DZ_), the contributions of each twin's PGS to their ADHD score (*g*_*tw*_), the contributions of the mother's and the father's PGS to their twins' ADHD scores (*g*_*m*_, *g*_*f*_), and the mean levels of PGS and ADHD scores. The fixed regression path of 0.5 between the parent's and twin's PGS represents the transmission of exactly half of parental autosomal DNA from parent to offspring, while the parental PGS correlation, *r*_*a*_, provides flexibility to explain parent‐child PGS correlations that deviate from 0.5 (Torvik et al., 2022). In turn, the parent‐child PGS correlations were used on a par with parent‐parent PGS correlation to estimate *r*_*a*_.

All these parameters except *r*_*MZ*_ and *r*_*DZ*_ were constrained to equality in MZ and DZ families and were estimated using full information maximum likelihood (FIML) that allows missing data (25% of all data points, no formal imputation procedure was performed). The contributions of each PGS to the twins' ADHD scores were standardized ($g_{tw}^{\prime }$, $g_{m}^{\prime }$, $g_{f}^{\prime }$). Additionally, we calculated the percentage of ADHD scores' variance explained by all PGS ($R_{\text{total}}^{2}$), as well as by direct genetic transmission and genetic nurture ($R_{gt}^{2}$, $R_{gn}^{2}$). The variance explained by direct genetic transmission, $R_{gt}^{2},$ was calculated as the amount of the variance explained by all polygenic scores in the model after the direct effects of parental polygenic score effects were removed. Then, the variance related to genetic nurture, $R_{gn}^{2}$, was computed as $R_{\text{total}}^{2}-R_{gt}^{2}$. Essentially, $R_{gn}^{2}$ represents the variance that is explained by any mechanisms involving parental PGS effects, including the effects of non‐transmitted parental alleles, as well as passive gene‐environment correlation. It is worth noting that parental and child's PGS effects can be estimated with opposite signs, meaning that the gene‐environment correlation could contribute negatively to $R_{\text{total}}^{2}$. Further details of the model are provided in Supporting Information S1.

Eight transmission models were tested, one for each combination of PGS (ADHD‐PGS or EA‐PGS), ADHD dimension (hyperactivity or inattention), and period (early childhood or primary school). Each model's fit was indexed by the Comparative Fit Index (CFI), Tucker‐Lewis Index (TLI), Root Mean Square Error of Approximation (RMSEA), as well as by the chi‐square test of the difference between the log‐likelihood of the target and saturated models (Likelihood Ratio Test, LRT). To test statistical significance of the model parameters ($g_{tw}^{\prime }$, $g_{m}^{\prime }$, $g_{f}^{\prime }$, $R_{\text{total}}^{2}$, $R_{gt}^{2}$, $R_{gn}^{2}$), we run bootstrap with 10,000 replications to compute the parameters' confidence intervals (CI). Testing six parameters within eight models posed a risk of Type I error, however, the models and parameters were not completely independent because (1) $R_{\text{total}}^{2}$, $R_{gt}^{2}$, $R_{gn}^{2}$ were computed from $g_{tw}^{\prime }$, $g_{m}^{\prime }$, $g_{f}^{\prime }$ and (2) hyperactivity and inattention were correlated within and between developmental periods. Therefore, in addition to a standard 95% CI, we chose to compute 99.2% CI that should account for the testing of three independent PGS effects ($g_{tw}^{\prime }$, $g_{m}^{\prime }$, $g_{f}^{\prime }$) by two PGS (ADHD‐ and EA‐PGS). Parameter was deemed significant when its CI excluded zero.

The data analysis was performed in the R statistical environment (R Core Team, 2022), and the OpenMx package was used for SEM (Neale et al., 2016). The full code of the data analyses is available on the first author's Github page: https://ivanvoronin.github.io/html/ADHD_intergenerational_JCPPA.html.

## RESULTS

### Preliminary analyses

#### Descriptive statistics and covariation of hyperactivity and inattention symptoms

Table 1 shows descriptive statistics for hyperactivity and inattention scores in early childhood and primary school for the total sample and by sex. Hyperactive symptoms were higher in early childhood (*M* = 0.83, *SD* = 0.40), in mother reports, than in primary school (*M* = 0.45, *SD* = 0.45), in teacher reports, *t* (696) = 19.92, *p* < 0.001. The opposite was noted for inattention (*M* = 0.63, *SD* = 0.35 in early childhood, *M* = 0.77, *SD* = 0.55 in primary school, *t* (696) = −6.45, *p* < 0.001). Boys were rated as more hyperactive and inattentive in early childhood and primary school; accordingly, further analyses controlled for sex differences. Hyperactivity and inattention symptoms were substantially correlated within each developmental period (*r* = 0.70, *p* < 0.001) and moderately across periods (*r* = 0.24–0.31, *p*_*s*_ < 0.001).

**TABLE 1:** Descriptive statistics of hyperactivity and inattention (both twins).

	*N*	All	Female	Male
		*M* (*SD*)	*M* (*SD*)	*M* (*SD*)
Early childhood				
1. Hyperactivity	724	0.83 (0.40)	0.80** (0.40)	0.87** (0.39)
2. Inattention	724	0.63 (0.35)	0.59*** (0.34)	0.67*** (0.36)
Primary school				
3. Hyperactivity	722	0.45 (0.45)	0.31*** (0.36***)	0.60*** (0.49***)
4. Inattention	722	0.77 (0.55)	0.62*** (0.50**)	0.92*** (0.56**)

Abbreviations: *M*, mean; SD, standard deviation.

*t*‐test of sex differences: *p < 0.05, **p < 0.01, ***p < 0.001.

Correlations between mothers' and fathers' PGSs were low and non‐significant (*r* = 0.07–0.08, *p* = 0.17–0.20, Table S3). The correlations between parents' and their twins' PGS were all above the expected 0.5 (*r* = 0.52–0.59, *p* = 0.02–0.55, Table S3), leaving a possibility of assortative mating and unaccounted population stratification. A moderate negative correlation between ADHD‐PGS and EA‐PGS was observed (*r* = −0.26, *p* < 0.001). Associations between parents' and twins' PGS (adjusted for ancestry/population stratification) and twins' ADHD dimensions are presented in Table 2. The correlations were generally low and variable across PGSs, family members (twins, mother, and father), developmental periods (early childhood and primary school), and ADHD dimensions. None of the ADHD‐PGS correlated with ADHD symptoms in early childhood; only child's ADHD‐PGS was modestly associated with primary school hyperactivity and inattention. By contrast, children's EA‐PGS was associated with hyperactivity, and especially inattention in early childhood and primary school. Parents' EA‐PGS modestly correlated with child's hyperactivity and inattention, mostly in primary school.

**TABLE 2:** Correlations between polygenic scores and the measures of hyperactivity and inattention.

	Child		Mother		Father	
	*r*	95% CI	*r*	95% CI	*r*	95% CI
ADHD PGS						
Early childhood						
Hyperactivity	0.025	[−0.048; 0.098]	0.058	[−0.042; 0.157]	0.010	[−0.096; 0.114]
Inattention	−0.024	[−0.097; 0.049]	0.020	[−0.080; 0.120]	−0.042	[−0.146; 0.063]
Primary school						
Hyperactivity	**0.095**	[0.022; 0.167]	−0.022	[−0.121; 0.077]	0.031	[−0.074; 0.135]
Inattention	**0.109**	[0.036; 0.180]	0.038	[−0.061; 0.137]	0.017	[−0.088; 0.121]
EA PGS						
Early childhood						
Hyperactivity	**−0.124**	[−0.195; −0.051]	**−0.105**	[−0.202; −0.005]	−0.055	[−0.159; 0.050]
Inattention	**−0.076**	[−0.148; −0.003]	−0.098	[−0.196; 0.002]	0.022	[−0.083; 0.127]
Primary school						
Hyperactivity	**−0.144**	[−0.215; −0.072]	−0.035	[−0.133; 0.065]	**−0.110**	[−0.212; −0.006]
Inattention	**−0.237**	[−0.305; −0.167]	**−0.184**	[−0.278; −0.087]	**−0.143**	[−0.244; −0.039]

*Note*: The statistically significant (*p* < 0.05) correlations are in bold.

Children's ADHD‐PGS more strongly correlated with their hyperactivity and inattention than their parents' PGS, suggesting direct genetic transmission. Alternatively, parents' EA‐PGS were significantly associated with ADHD dimensions almost as strongly as the children's EA‐PGS, which could point to genetic nurture.

### Intergenerational transmission

**FIGURE 2:**
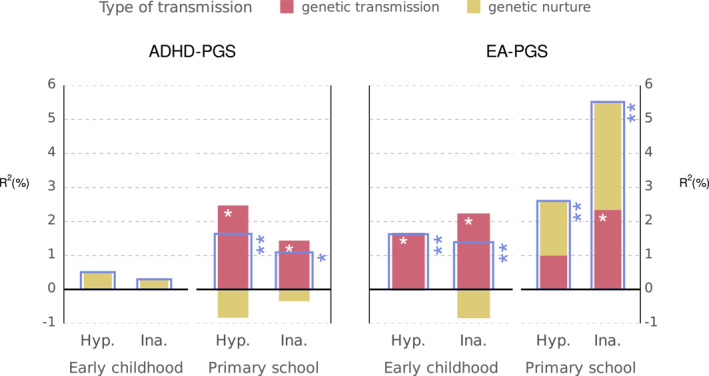
Percentage of ADHD symptoms' variance explained by direct genetic and genetic nurture transmission in early childhood and primary school by ADHD‐PGS and EA‐PGS. Blue bars show total *R* ^2^. The white asterisks mark statistical significance of genetic transmission and genetic nurture variance, blue asterisks mark statistical significance of total variance (*p < 0.05, **p < 0.008). Hyp., hyperactivity; ina., inattention.

**FIGURE 3:**
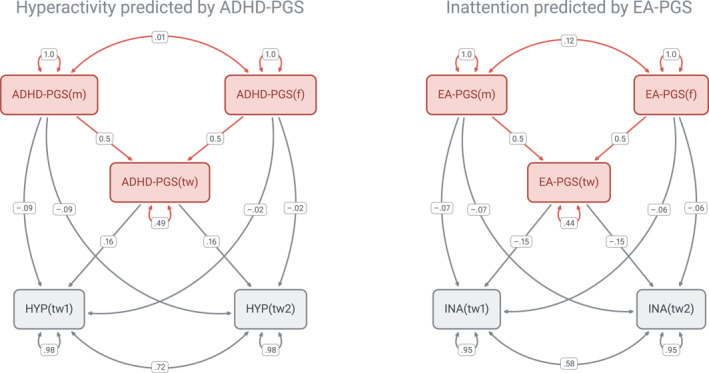
Standardized parameter estimates in the transmission model with ADHD‐PGS predicting hyperactivity in primary school (left) and EA‐PGS predicting inattention in primary school (right). Only MZ families are depicted. f, father; HYP, hyperactivity; INA, inattention; m, mother; tw, twin.

The intergenerational model (Figure 1) was tested for each combination of genetic propensity (ADHD‐PGS or EA‐PGS), developmental period (early childhood or primary school) and ADHD dimension (hyperactivity or inattention). The model provided a good fit to the data in all cases (Table S4). A summary of the transmission models' results is presented in Figure 2, which shows the percentages of variance explained by the PGS overall ($R_{\text{total}}^{2}$), genetic transmission ($R_{gt}^{2}$), and genetic nurture ($R_{gn}^{2}$). The estimates of direct PGS contributions ($g_{tw}^{\prime },g_{m}^{\prime },g_{f}^{\prime }$), and the variance explained ($R_{\text{total}}^{2}$, $R_{gt}^{2}$, $R_{gn}^{2}$) are presented in Table 3 (ADHD‐PGS) and Table 4 (EA‐PGS). Tables S5 and S6 in Supporting Information show the same estimates but with the extended confidence interval. Figure 3 presents the depiction of the model with ADHD‐PGS predicting hyperactivity in primary school and EA‐PGS predicting inattention in primary school, populated with parameter estimates.

**TABLE 3:** Estimates of direct polygenic score effects and variance explained by **ADHD‐PGS**.

	Hyperactivity		Inattention	
	est.	95% CI	est.	95% CI
Early childhood				
Direct contributions				
Child's ADHD‐PGS, $g_{tw}^{\prime }$	0.016	[−0.091; 0.116]	0.002	[−0.100; 0.099]
Mother's ADHD‐PGS, $g_{m}^{\prime }$	0.056	[−0.056; 0.161]	0.000	[−0.106; 0.105]
Father's ADHD‐PGS, $g_{f}^{\prime }$	−0.038	[−0.164; 0.092]	−0.055	[−0.180; 0.068]
Explained variance				
Total (%)	0.5	[0.0; 1.6]	0.3	[0.0; 1.0]
Genetic transmission (%)	0.0	[0.0; 0.3]	0.0	[0.0; 0.0]
Genetic nurture (%)	0.5	[−0.6; 2.6]	0.3	[−0.6; 1.9]
Primary school				
Direct contributions				
Child's ADHD‐PGS, $g_{tw}^{\prime }$	0.157**	[0.055; 0.249]	0.120*	[0.027; 0.217]
Mother's ADHD‐PGS, $g_{m}^{\prime }$	−0.089	[−0.187; 0.006]	−0.026	[−0.128; 0.072]
Father's ADHD‐PGS, $g_{f}^{\prime }$	−0.015	[−0.114; 0.097]	−0.009	[−0.110; 0.099]
Explained variance				
Total (%)	1.6**	[0.3; 3.4]	1.1*	[0.1; 2.3]
Genetic transmission (%)	2.5*	[0.3; 6.2]	1.4*	[0.1; 4.7]
Genetic nurture (%)	−0.8	[−3.4; 1.1]	−0.3	[−2.6; 1.1]

Abbreviation: est., estimate.

*p < 0.05, **p < 0.008; statistically significant values (*p* < 0.008) are in bold.

**TABLE 4:** Estimates of direct polygenic score effects and variance explained by **EA‐PGS**.

	Hyperactivity		Inattention	
	est.	95% CI	est.	95% CI
Early childhood				
Direct contributions				
Child's EA‐PGS, $g_{tw}^{\prime }$	−0.129*	[−0.226; −0.030]	−0.150**	[−0.251; −0.047]
Mother's EA‐PGS, $g_{m}^{\prime }$	−0.001	[−0.100; 0.090]	0.013	[−0.095; 0.116]
Father's EA‐PGS, $g_{f}^{\prime }$	0.004	[−0.103; 0.108]	0.100*	[0.001; 0.209]
Explained variance				
Total (%)	1.6**	[0.3; 3.0]	1.4**	[0.2; 3.2]
Genetic transmission (%)	1.7	[0.1; 5.1]	2.2*	[0.2; 6.3]
Genetic nurture (%)	0.0	[−2.6; 2.0]	−0.8	[−3.8; 1.1]
Primary school				
Direct contributions				
Child's EA‐PGS, $g_{tw}^{\prime }$	−0.100*	[−0.196; −0.002]	−0.153**	[−0.253; −0.051]
Mother's EA‐PGS, $g_{m}^{\prime }$	0.020	[−0.083; 0.122]	−0.070	[−0.165; 0.028]
Father's EA‐PGS, $g_{f}^{\prime }$	−0.091	[−0.193; 0.016]	−0.060	[−0.173; 0.064]
Explained variance				
Total (%)	2.6**	[0.7; 4.9]	5.5**	[2.8; 8.3]
Genetic transmission (%)	1.0	[0.0; 3.8]	2.3*	[0.3; 6.4]
Genetic nurture (%)	1.6	[−1.3; 4.8]	3.2	[−1.4; 7.4]

Abbreviation: est., estimate.

*p < 0.05, **p < 0.008; statistically significant values (*p* < 0.008) are in bold.

#### Intergenerational transmission of genetic propensities for ADHD

The contributions of ADHD‐PGS to children's ADHD dimensions are shown in Table 3 and the left panel of Figure 2. In early childhood, neither the parents' nor the children's ADHD‐PGS contributed to children's hyperactivity and inattention. In primary school, children's ADHD‐PGS significantly predicted both hyperactivity ($g_{tw}^{\prime }$ = 0.16, *p* < 0.008, Table S5) and inattention ($g_{tw}^{\prime }$ = 0.12, *p* < 0.05), whereas the direct contributions of parents' ADHD‐PGS were low and non‐significant. Together, children's and parents' ADHD‐PGS accounted for 1.6% of the variance in hyperactivity and 1.1% in inattention in primary school. Direct genetic transmission was identified as the sole significant source of variance in ADHD dimensions, accounting for 2.5% and 1.4% of the variance in hyperactivity and inattention, respectively (*p* < 0.05). Direct genetic transmission exceeded the total explained variance due to the non‐significant, but negative estimates of genetic nurture (Figure 2, left panel).

#### Intergenerational transmission of genetic propensities for education attainment

The contributions of parents' and children's EA‐PGS to hyperactivity and inattention are presented in Table 4 and in the right panel of Figure 2. By contrast to ADHD‐PGS, children's EA‐PGS significantly predicted hyperactivity and inattention in both early childhood and primary school, with estimates of $g_{tw}^{\prime }$ ranging between −0.10 and −0.15 (*p* < 0.05 for hyperactivity and *p* < 0.008 for inattention, Table 4). Parents' EA‐PGS contributions were non‐significant, except for fathers' EA‐PGS modestly predicting children's inattention in early childhood ($g_{f}^{\prime }$ = 0.10, *p* < 0.05). Together, children's, mothers', and fathers' EA‐PGS accounted for 1.6% and 1.4% of the variance in hyperactivity and inattention in early childhood, but 2.6% and 5.5%, respectively in primary school (all *p* < 0.008, Table S6).

Direct genetic transmission significantly accounted for 1.0% and 2.3% of the variance in hyperactivity and inattention, respectively, in primary school (*p* < 0.05, right panel of Figure 2, Table 4). The contribution of genetic nurture to inattention in early childhood was estimated to be zero and non‐significant. In primary school, genetic nurture accounted for 1.6% and 3.2% in hyperactivity and inattention respectively, but the estimates were nonsignificant when compared to the full model without genetic nurture effects (*p* = 0.36 and 0.41).

## DISCUSSION

This study aimed to test a novel integrated model of intergenerational transmission of childhood ADHD symptoms that distinguished direct genetic transmission from genetic nurture, while also controlling for assortative mating and population stratification. Findings revealed that measured genetic propensities for ADHD and educational attainment—in the form of PGS—in children and their parents significantly predicted children's hyperactivity and inattention symptoms in early childhood and primary school (ADHD‐PGS only in primary school). Overall, the predictions were modest and mainly operated through direct genetic transmission, but these associations varied across developmental periods, PGS, and ADHD dimensions. Specifically, more conclusive findings were evidenced for primary school than for early childhood ADHD symptoms, and more so for EA‐PGS than for ADHD‐PGS. Both primary school hyperactivity and inattention were predicted directly by child's own PGS, and especially inattention by the EA‐PGS. There was also a signal of genetic nurture for EA‐PGS in primary school in terms of explained variance, which did not reach statistical significance. These findings expand our understanding of the intergenerational transmission of ADHD symptoms in several ways.

First, the present study confirmed the capacity of known PGS to predict the two main dimensions of ADHD (i.e., hyperactivity and inattention), as measured continuously in a population‐based sample of children. Given the known heritability of ADHD manifestations, the magnitude of the predictions was modest, but in line with previous genomic research with PGS (Agnew‐Blais et al., 2022; de Zeeuw et al., 2020; Martin et al., 2023; Pingault et al., 2022; Selzam et al., 2019). As the power of future GWAS increases and new estimations of PGS can be derived, their prediction value will likely increase (Raffington et al., 2020). The proposed structural equation model also confirmed that the transmission of genetic risk for ADHD was mainly channeled through genetic variants that are directly passed on from parents to the child's genotype (Axelrud et al., 2023; de Zeeuw et al., 2020; Frach et al., 2023; Martin et al., 2023; Pingault et al., 2022; Selzam et al., 2019). This was expected, but controlling for the alternate transmission pathway (i.e., genetic nurture) and the correlation between parental PGS (Boomsma et al., 2010; Hugh‐Jones et al., 2016; Nordsletten et al., 2016), the present study provides a more stringent estimate of this direct genetic pathway. Although we found no clear evidence of assortative mating, parent‐offspring correlations were above 0.5, some significantly. Previous studies reported modest assortative mating relative to EA‐PGS (*r* = 0.011 Agnew‐Blais et al., 2022; Hugh‐Jones et al., 2016). Clearly, PGS only provides an approximation of genetic effects (Torvik et al., 2022), and assortative mating should be investigated further.

There are many reasons why the PGS were more predictive of ADHD symptoms in primary school than in early childhood. For instance, ADHD dimension assessments may be less valid in preschool than in primary school, as they were repeatedly reported by the mother in early childhood, whereas different teachers rated the child in primary school, thus providing more independent perspectives on the child's symptoms. Furthermore, given the importance of attention and behavioral control in learning, primary school learning contexts are more likely to reveal hyperactivity and inattention symptoms than preschool contexts (Murray et al., 2018), and teachers are better positioned than mothers to reliably compare the child's behaviors to the classroom/school norm and a larger sample of children. However, twin studies consistently showed high heritability of ADHD symptoms in both preschool (Price et al., 2005) and the school years (Bergen et al., 2007; Chang et al., 2013), which may be less efficiently captured in PGS derived from GWASs performed on a combined sample of children and adults.

The present study measured hyperactivity separately from inattention symptoms, thus enabling the examination of their distinct genetic etiology. However, the statistical estimates of these differences did not reach significance. Overall, the EA‐PGSs performed better than ADHD‐PGSs in predicting both ADHD dimensions. The superior predictive validity of EA GWAS for a variety of human capital outcomes has been well documented (Barth et al., 2018; Belsky et al., 2016). This is likely due to the well‐powered discovery GWAS provided by the huge sample on which EA‐PGS was based. At the same time, while ADHD‐PGS was more strongly associated with hyperactivity, EA‐PGSs predicted both dimensions, but especially inattention in primary school. This is consistent with the known negative association between inattention and educational outcomes (Gray et al., 2017; Pingault et al., 2011), partially due to pleiotropic genetic effects (genetic variants associated with multiple traits, Greven, Harlaar, et al., 2011). Future studies should examine this inattentive pathway further, specifically the extent to which inattention mediates the association between genetic propensity of the EA‐PGS and various academic outcomes.

Our model evidenced direct genetic transmission as the main mechanism of intergenerational transmission of ADHD, in line with previous research using PGS (Agnew‐Blais et al., 2022; de Zeeuw et al., 2020; Demontis et al., 2023; Pingault et al., 2022; Selzam et al., 2019). We also noted an ostensible part of the variance of inattention in primary school was channeled by EA‐PGS, seemingly through genetic nurture transmission. However, despite non‐trivial effect size (3.2%) for a PGS, this indirect effect did not reach statistical significance. Such a pathway had not been previously detected in PGS studies, but is consistent with a family study in which a significant genetic nurture transmission for hyperactivity, inattention, and other externalizing problems was detected (Eilertsen et al., 2022). Future studies with greater statistical power should more robustly test the possibility of genetic nurture effects for these phenotypes. Incidentally, as in the present study, Eilertsen et al. (2022) also detected negative covariance between direct (child's) and indirect (parents') genetic effects for inattention. Considering the possibility of negative gene‐environment correlation is important because (1) it is potentially amenable through an intervention, (2) it contributes toward the decrease of the outcome's heritability, that is, a drive for intergenerational discontinuity. Our study shows how this effect can be brought to light and estimated with the PGS data.

### Strengths and limitations

In this study we proposed and tested a novel model of intergenerational transmission that accommodated the data of twin families. The model provides a nuanced representation of direct and environmentally mediated (genetic nurture) genetic effects on children's traits. The model allows incomplete data and takes into account genetic similarity between parents that arise from assortative mating and unaccounted population stratification. Using structural equation modeling, the model can be adapted to other family structures (e.g., singleton families, extended pedigree design), and extended to include measured environmental variables that are hypothesized to partially or entirely explain genetic nurture effects. Finally, our model estimates the direct PGS effects, in the form of regression coefficients, as well as percent of the variance explained by all PGS and by two alternative transmission pathways that provides a valuable insight into the composition of the individual differences of ADHD.

The main limitation of our study is the lack of statistical power due to missing parental genotypes, which are essential for the estimation of direct parental effects, and more generally due to the relatively small number of families for this type of studies. For this reason, even the genetic nurture estimate of 3.2% for inattention in primary school was not statistically significant. Further research with higher statistical power is needed. Then, the phenotypic measures used in the present study could not fully examine potential reporter bias because hyperactivity and inattention symptoms were reported by different raters across developmental periods (mothers in early childhood and by teachers in primary school). Additionally, alternative approaches to the phenotypic scoring are possible, such as CFA. We thus estimated in sensitivity analysis factor scores of hyperactivity and inattention in early childhood and primary school in a one‐factor CFA model. These scores strongly correlated with the scores that we used in the study (*r* = 0.88–0.96), which indicates that it is unlikely that the selected analytical approach used to operationalize the phenotypes negatively affected the results. That being said, we could have aggregated the ADHD scores over two developmental periods, potentially revealing a more robust genetic effect if those are mainly driven by stable patterns of individual differences. However, this would come at the expense of developmental specificity. Future studies with more statistical power could account for developmental stability and specificity, as well as the overlap between hyperactivity and inattention dimensions using multilevel CFA.

Some shortcomings of our study come from the specific aspects of methods and methodology that we chose to use. In particular, the PGS, as a genetic predictor, only captures genetic risk tied to a specific phenotype and measures it with limited precision. For this reason, the transmission effects, as well as the subtle effects like assortative mating and unaccounted population stratification, may not be captured in full in a study with PGS. Then, it has been demonstrated recently that the genetic nurture effect detected in the intergenerational transmission research may result from the differences between family lineages accumulated across generations (dynastic effect), rather than from the processes within a family (Nivard et al., 2024). Consequently, an integrative approach is warranted in the research of intergenerational transmission.

## CONCLUSION

Measured genetic propensities toward ADHD and education modestly contribute to individual differences in hyperactivity and inattention, and this contribution is more apparent in primary school than in early childhood. Direct genetic transmission was identified as a primary mechanism of intergenerational transmission of both genetic predictors. While no significant genetic nurture transmission was found, the results warrant further investigation in more powered samples. The model developed and applied in this study can be a useful tool for the research of intergenerational transmission of ADHD and other traits.

## AUTHOR CONTRIBUTIONS

**Ivan Voronin**: Conceptualization; data curation; formal analysis; investigation; methodology; software; visualization; writing—original draft; writing—review and editing. **Isabelle Ouellet‐Morin**: Conceptualization; funding acquisition; methodology; project administration; resources; supervision; writing—original draft; writing—review and editing. **Amelie Petitclerc**: Funding acquisition; project administration; resources; writing—review and editing. **Geneviève Morneau‐Vaillancourt**: Data curation; writing—original draft; writing—review and editing. **Mara Brendgen**: Project administration; writing—original draft; writing—review and editing. **Ginette Dionne**: Project administration; writing—original draft; writing—review and editing. **Frank Vitaro**: Project administration; writing—original draft; writing—review and editing. **Michel Boivin**: Conceptualization; funding acquisition; investigation; methodology; project administration; resources; supervision; writing—original draft; writing—review and editing.

## CONFLICT OF INTEREST STATEMENT

No conflict of interest.

## ETHICAL CONSIDERATIONS

Parental and child informed consent was obtained from the ethics review board at Université Laval, Quebec.

## SUPPORTING INFORMATION

The full code of data analysis is available on the first author's Github page: https://ivanvoronin.github.io/html/ADHD_intergenerational_JCPP.html. Figures 1, 2, and 3 are published on OSF (https://osf.io/7zs2y/) under a Creative Commons license (CC BY‐NC‐SA 4.0).

## Supporting information

Supporting Information S1

## Data Availability

The sample of the current study was drawn from the dataset that is not publicly available because the informed consent obtained from QNTS participants prohibits the distribution of the data through any third party maintained public repository. However, the data may be provided upon request, see http://www.gripinfo.ca/grip/public/www/Etudes/en/dadprocedures.asp for more information. The details on the data from the QNTS are available on the study's website (https://maelstrom‐research.org/study/ejnq).
