# Supplementary material for: Intergenerational transmission of genetic risk for hyperactivity and inattention. Direct genetic transmission or genetic nurture?
Source: JCPP Adv. 2024 Mar 4;4(2):e12222. doi: 10.1002/jcv2.12222 (PMC11143957; doi:10.1002/jcv2.12222)
Supplement: Supplementary file 1 — Supporting Information S1 [file JCV2-4-e12222-s001.pdf]

Intergenerational transmission of genetic risk for hyperactivity and inattention. Genetic transmission or genetic nurture?

Ivan Voronin, Isabelle Ouellet-Morin, Amélie Petitclerc, Geneviève Morneau-Vaillancourt, Mara Brendgen, Ginette Dione, Frank Vitaro, Michel Boivin

Supporting Information

Contents:

- **Appendix S1.** Supplementary Methods
  - Genotyping, quality control and polygenic score computation
  - Implementation of the transmission model
  - Derivation of $R^2{}_{gt}$ and $R^2{}_{gn}$
  - References
- **Table S1.** The summary of available genotype data in QNTS families
- **Table S2.** Sociodemographic characteristics of the sample, comparison of participants that were included and excluded from the study
- **Table S3.** Correlations between family members' PGS
- **Table S4.** Fit statistics of transmission model
- **Table S5.** Estimates of direct polygenic score effects and variance explained by ADHD PGS with 99.2% confidence interval
- **Table S6.** Estimates of direct polygenic score effects and variance explained by ADHD PGS with 99.2% confidence interval

Appendix S1. Supplementary Methods

Genotyping, quality control and polygenic score computation

Genotyping was conducted using the Infinium PsychArray-24 v1.3 BeadChip. The quality control (QC) of genetic data was conducted in PLINK v1.90b5.3, PLINK v1.90b6.7 (Chang et al., 2015), and R v3.4.3.

Pre-imputation QC of genotype data consisted of the following steps:

1. Removal of SNPs with call rates <98% or a minor allele frequency (MAF) <1%
2. Removal of individuals with genotyping rates <95%
3. Removal of sex mismatches
4. Removal of genetic duplicates
5. Removal of cryptic relatives with pi-hat>=12.5
6. Removal of genetic outliers with a distance from the mean of >4 SD in the first eight multidimensional scaling (MDS) ancestry components
7. Removal of individuals with a deviation of the autosomal or X-chromosomal heterozygosity from the mean >4 SD
8. Removal of non-autosomal variants
9. Removal of SNPs with call rates <98% or a MAF <5% or Hardy-Weinberg Equilibrium (HWE) test p-values <1×10-3
10. Removal of A/T and G/C SNPs
11. Update of variant IDs and positions to the IDs and positions in the 1000 Genomes Phase 3 reference panel
12. Alignment of alleles to the reference panel
13. Removal of duplicated variants and variants not present in the reference panel

These steps of QC were performed separately on the data of Quebec Newborn Twin Study (QNTS) and Quebec Longitudinal Study of Child Development (QLSCD), then the data were combined in a consolidated genotype panel.

For the calculation of ancestry components used to determine genetic outliers, pre-imputation genotype data were used. Additional variant filtering steps were: removal of variants with a MAF <0.05 or HWE p value <0.001; removal of variants mapping to the extended MHC region (chromosome 6, 25-35 Mbp) or to a typical inversion site on chromosome 8 (7-13 Mbp); linkage disequilibrium (LD) pruning (command --indep-pairwise 200 100 0.2). Next, the pairwise identity-by-state (IBS) matrix of all individuals was calculated using the command genome on the filtered genotype data. Multidimensional

scaling (MDS) analysis was performed on the IBS matrix using the eigendecomposition-based algorithm in PLINK v1.90b6.7 (QLSCD) and PLINK v1.90b5.2 (QNTS).

Imputation was conducted using SHAPEIT v2 (r837) (Delaneau, Zagury, & Marchini, 2013), IMPUTE2 v2.3.2 (Howie, Donnelly, & Marchini, 2009), and the 1000 Genomes Phase 3 reference panel. After imputation, variants with a MAF <1%, an HWE test p<1×10-6, and an INFO metric <0.8 were removed. In total, imputed genetic data were available for 443 individuals in the QNTS sample (including both DZ twins for 126 families) and for 816 individuals in the QLSCD sample. Variants before QC: 588,952; variants after QC and imputation: 8,407,807.

The polygenic scores were computed using the SNP summary statistics from respective GWAS: ADHD GWAS (Demontis et al., 2019) and GWAS for educational attainment (Okbay et al., 2022). The summary statistics were adjusted using Bayesian approach implemented in PRS-CS software (Ge, Chen, Ni, Feng, & Smoller, 2019, the global shrinkage parameter phi = 0.01) separately for each autosome (the sex chromosomes were not analyzed). The negative SNP effects were flipped, and then the summary statistics across the chromosomes were combined into a single file. At the last step of computation, the polygenic score was computed using PLINK v1.90b6.21.

The population stratification was controlled for using the factor scores on the first ten principal components of the genetic relatedness matrix (Price et al., 2006) derived from a subsample of genetically unrelated individuals in the combined QNTS/QLSCD genotype panel. The subsample of unrelated individuals was determined using KING 2.3.0 (Manichaikul et al., 2010).

Implementation of the transmission model

The analytic strategy used to distinguish direct genetic transmission and genetic nurture of ADHD risk relied on the structural equation modeling approach (SEM, Loehlin, 2004). Typically, a model in SEM is defined as a set of linear equations that describe relationships between (measured and unmeasured) variables in the model. Parameters of the model are estimated by minimizing the difference between model-implied and empirical covariance matrices (least squares optimization). Alternatively, the estimates can be found by maximizing the likelihood of the data conditioned on the multivariate distribution implied by the model (likelihood-based optimization). Both approaches approximate mean and

covariance structure observed in the data. Our analysis relied on the Full Information Maximum Likelihood (FIML) estimation that provided the best account for missing data.

The MZ and DZ covariance structures were specified via RAM-approach (Reticular Action Model) that portrays a covariance matrix as matrix algebra: $\Sigma = F \times (I - A)^{-1} \times S \times (I - A)^{-1T} \times F^T$ (Loehlin, 2004; McArdle & McDonald, 1984). If t is the total number of variables in a model and m is the number of measured variables, F is $m \times t$ matrix that selects measured variables (a filter matrix), A is $t \times t$ matrix of one-way (asymmetric) relationships, S is $t \times t$ matrix of variances and covariances (symmetric relationships), and I is $t \times t$ identity matrix. Since all variables in the transmission model were measured, we did not use the filter matrix and defined MZ and DZ covariance structures as following:

$$\Sigma_{MZ} = (I_{5\times5} - A_{MZ})^{-1} \times S_{MZ} \times (I_{5\times5} - A_{MZ})^{-1T},$$
$$\Sigma_{DZ} = (I_{6\times6} - A_{DZ})^{-1} \times S_{DZ} \times (I_{6\times6} - A_{DZ})^{-1T},$$

where Σ_{MZ}, $I_{5\times5}$, A_{MZ}, and S_{MZ} are 5×5 matrices and Σ_{DZ}, $I_{6\times6}$, A_{DZ}, and S_{DZ} are 6×6 matrices:

$$S_{MZ} = \begin{bmatrix} V_{PGS} & r_a V_{PGS} & \frac{(1+r_a)}{2} V_{PGS} & 0 & 0 \\ r_a V_{PGS} & V_{PGS} & \frac{(1+r_a)}{2} V_{PGS} & 0 & 0 \\ \frac{(1+r_a)}{2} V_{PGS} & \frac{(1+r_a)}{2} V_{PGS} & V_{PGS} & 0 & 0 \\ 0 & 0 & 0 & E_{ADHD} & Cov_{ADHD,MZ} \\ 0 & 0 & 0 & Cov_{ADHD,MZ} & E_{ADHD} \end{bmatrix}$$

$$A_{MZ} = \begin{bmatrix} 0 & 0 & 0 & 0 & 0 \\ 0 & 0 & 0 & 0 & 0 \\ 0 & 0 & 0 & 0 & 0 \\ g_m & g_f & g_{tw} & 0 & 0 \\ g_m & g_f & g_{tw} & 0 & 0 \end{bmatrix} \quad I_{5\times5} = \begin{bmatrix} 1 & 0 & 0 & 0 & 0 \\ 0 & 1 & 0 & 0 & 0 \\ 0 & 0 & 1 & 0 & 0 \\ 0 & 0 & 0 & 1 & 0 \\ 0 & 0 & 0 & 0 & 1 \end{bmatrix}$$

$$S_{DZ} = \begin{bmatrix} V_{PGS} & r_a V_{PGS} & \frac{(1+r_a)}{2} V_{PGS} & \frac{(1+r_a)}{2} V_{PGS} & 0 & 0 \\ r_a V_{PGS} & V_{PGS} & \frac{(1+r_a)}{2} V_{PGS} & \frac{(1+r_a)}{2} V_{PGS} & 0 & 0 \\ \frac{(1+r_a)}{2} V_{PGS} & \frac{(1+r_a)}{2} V_{PGS} & V_{PGS} & \frac{(1+0.5r_a)}{2} V_{PGS} & 0 & 0 \\ \frac{(1+r_a)}{2} V_{PGS} & \frac{(1+r_a)}{2} V_{PGS} & \frac{(1+0.5r_a)}{2} V_{PGS} & V_{PGS} & 0 & 0 \\ 0 & 0 & 0 & 0 & E_{ADHD} & Cov_{ADHD,DZ} \\ 0 & 0 & 0 & 0 & Cov_{ADHD,DZ} & E_{ADHD} \end{bmatrix}$$

$$A_{DZ} = \begin{bmatrix} 0 & 0 & 0 & 0 & 0 & 0 \\ 0 & 0 & 0 & 0 & 0 & 0 \\ 0 & 0 & 0 & 0 & 0 & 0 \\ 0 & 0 & 0 & 0 & 0 & 0 \\ g_m & g_f & g_{tw} & 0 & 0 & 0 \\ g_m & g_f & 0 & g_{tw} & 0 & 0 \end{bmatrix} \quad I_{6\times 6} = \begin{bmatrix} 1 & 0 & 0 & 0 & 0 & 0 \\ 0 & 1 & 0 & 0 & 0 & 0 \\ 0 & 0 & 1 & 0 & 0 & 0 \\ 0 & 0 & 0 & 1 & 0 & 0 \\ 0 & 0 & 0 & 0 & 1 & 0 \\ 0 & 0 & 0 & 0 & 0 & 1 \end{bmatrix}$$

The matrices A_{MZ} and A_{DZ} included three estimated parameters that represented direct contributions of the polygenic score to the individual differences in the ADHD symptom: g_m - contribution of mother's polygenic score, g_f - contribution of father's polygenic score, and g_{tw} - contribution of twin's polygenic score. These parameters were estimated conditionally on each other in the model, therefore g_m and g_f tap into the parental genotype effects that bypass a child's genotype, or genetic nurture effects.

The matrices S_{MZ} and S_{DZ} included five parameters to model variance and covariance relationships: V_{PGS} - the variance of polygenic score, r_a - the correlation between mother's and father's polygenic scores that represented genetic similarity due to assortative mating and unaccounted population structure, E_{ADHD}, $Cov_{ADHD,MZ}$ and $Cov_{ADHD,DZ}$ - the variance and cross-twin covariance of the ADHD symptom that remained unaccounted for by the polygenic prediction in the model. The PGS covariance in the matrices S_{MZ} and S_{DZ} represented the structure of relationships between the PGS depicted on Figure 1. It operates under two assumptions: 1) the variances of the PGS of all family members are equal (V_{PGS}), and 2) each parent provides exactly half of their genotype to the child (fixed 0.5 paths from parents' to child's PGS).

In total, ten parameters were estimated in the model. To simplify the interpretation of results, we standardized the key parameters of the model: $g'_{tw} = g_{tw} \cdot \frac{SD_{PGS}}{SD_{ADHD}}, g'_m = g_m \cdot \frac{SD_{PGS}}{SD_{ADHD}}, g'_f = g_f \cdot \frac{SD_{PGS}}{SD_{ADHD}}, E'_{ADHD} = \frac{E_{ADHD}}{SD^2_{ADHD}}$, where SD_{PGS} and SD_{ADHD} are the standard deviations of the polygenic score and ADHD symptom estimated from the model. Therefore, g'_{tw}, g'_m, g'_f indicate how much of the standard deviation of the ADHD symptom corresponds to the difference of one standard deviation difference in the polygenic score of a twin, mother or father, respectively. The standardized variance E'_{ADHD} indicates the proportion of the variance that remained unexplained by the polygenic scores (in the range from 1, all variance unexplained, to 0, all variance explained).

To assess how much of the variance of ADHD is explained by genetic transmission, genetic nurture and in total, we computed three secondary parameters. First, $R^2{}_{total}$ was the total variance explained by the polygenic scores: $R^2{}_{total} = 1 - E'_{ADHD}$. Second, $R^2{}_{vt}$ was the variance explained by genetic transmission: $R^2{}_{gt} = 1 - E'_{ADHD,nogn}$, where $E'_{ADHD,nogn}$ is a standardized E_{ADHD} once the effects of parental polygenic scores (g_m and g_f) are removed from the A-matrix. The removal of the effects of parental polygenic scores from the formula limited the sources of the variance to those channeled exclusively through the child's genotype, making $R^2{}_{gt}$ the variance explained by the directly transmitted genetic risk. Finally, $R^2{}_{gn}$ was the variance explained by genetic nurture: $R^2{}_{gn} = R^2{}_{total} - R^2{}_{gt}$, reflecting the fact that the genetic transmission and genetic nurture variance add up to the total variance explained by the polygenic score.

Derivation of $R^2{}_{gt}$ and $R^2{}_{gn}$

In this section we are going to show how exactly $R^2{}_{gt}$ and $R^2{}_{gn}$ are derived from the parameter estimates in the transmission model. We are going to operate with the standardized estimates of PGS effects, g'_{tw}, g'_{m}, g'_{f}, as well as with the correlation between parental PGS, r_a. The full variance of all variables in the standardized solution is scaled to unity.

The RAM-specification of the model is equivalent to the path diagram specification where the contributions to the (co)variance of the variables are determined by Wright rules (e.g., see Balbona et al., 2021). As a SEM model, the transmission model includes two types of paths: one-way paths, or regression, and two-way paths, or variance, covariance and correlation. The total expected (co)variance is identified as a sum of the (co)variance contributed by all valid chains of paths that connect two variables in case of covariance or one variable with itself in case of variance. According to Wright rules, a valid chain travels upwards the one-way paths, then passes through exactly one two-way path, then travels down the one-way paths. All chains must be unique and two chains that travel across the same paths but in the opposite order are considered two different path chains. The contribution of each chain to the total (co)variance is defined as a product of parameter estimates on each path crossed by this chain.

Let us consider several examples of the valid chains in our model (Figure 1):

1) ADHD(tw1) -> PGS(tw) -> PGS(m) -> PGS(tw) -> ADHD(tw1) - this chain contributes $g_{tw} \cdot 0.5 \cdot V_{PGS} \cdot 0.5 \cdot g_{tw}$ to the total variance of the ADHD trait (ADHD(tw1)) through genetic transmission mechanism, exclusively via twin's PGS effect;

2) ADHD(tw1) -> PGS(m) -> ADHD(tw1) - this chain contributes $g_m \cdot V_{PGS} \cdot g_m$ to the variance through genetic nurture transmission via parent's PGS effect;
3) ADHD(tw1) -> PGS(tw) -> PGS(m) -> ADHD(tw1) - this chain contributes $g_{tw} \cdot 0.5 \cdot V_{PGS} \cdot g_m$ to the total variance through genetic nurture transmission via both twin's and parent's PGS effect, i.e. via gene-environment correlation.

Let us show how the genetic transmission and genetic nurture are derived using Wright rules. First, the variance contributed by genetic transmission, ${R^2}_{gt}$, is contributed by all chains that come to ADHD(tw) exclusively through twin's PGS effect, g_{tw}, and since all these chains pass through PGS(tw) with the full variance amounted to 1 in the standardized solution, the genetic transmission variance is computed as:

$${R^2}_{gt} = {g'}_{tw} \cdot 1 \cdot {g'}_{tw} = ({g'}_{tw})^2$$

Second, the variance contributed by genetic nurture transmission, ${R^2}_{gn}$, includes two types of chains: 1) the chains that pass exclusively through parent's PGS effects, 2) the chains that pass through both parent's and twin's PGS effects. The contribution of the former is computed as:

$$\begin{gathered} {g'}_m \cdot 1 \cdot {g'}_m + {g'}_f \cdot 1 \cdot {g'}_f + {g'}_m \cdot r_a \cdot {g'}_f + {g'}_f \cdot r_a \cdot {g'}_m = \\ = ({g'}_m)^2 + \left({g'}_f\right)^2 + 2 \cdot r_a \cdot {g'}_m \cdot {g'}_f \end{gathered}$$

The contribution of the latter (gene-environmental correlation) is computed as:

$$\begin{gathered} {g'}_m \cdot 1 \cdot 0.5 \cdot {g'}_{tw} + {g'}_{tw} \cdot 0.5 \cdot 1 \cdot {g'}_m + {g'}_m \cdot r_a \cdot 0.5 \cdot {g'}_{tw} + {g'}_{tw} \cdot 0.5 \cdot r_a \cdot {g'}_m + \\ + {g'}_f \cdot 1 \cdot 0.5 \cdot {g'}_{tw} + {g'}_{tw} \cdot 0.5 \cdot 1 \cdot {g'}_f + {g'}_f \cdot r_a \cdot 0.5 \cdot {g'}_{tw} + {g'}_{tw} \cdot 0.5 \cdot r_a \cdot {g'}_f = \\ = (1 + r_a) \cdot {g'}_m \cdot {g'}_{tw} + (1 + r_a) \cdot {g'}_f \cdot {g'}_{tw} = \\ = (1 + r_a) \cdot \left({g'}_m + {g'}_f\right) \cdot {g'}_{tw} \end{gathered}$$

The total variance explained by genetic nurture is:

$${R^2}_{gn} = ({g'}_m)^2 + \left({g'}_f\right)^2 + 2 \cdot r_a \cdot {g'}_m \cdot {g'}_f + (1 + r_a) \cdot \left({g'}_m + {g'}_f\right) \cdot {g'}_{tw}$$

Note that the contribution of the gene-environmental correlation turns negative when parental PGS effects $({g'}_m, {g'}_f)$ and twin's PGS effect $({g'}_{tw})$ have opposite signs.

References

Balbona, J. V., Kim, Y., & Keller, M. C. (2021). Estimation of Parental Effects Using Polygenic Scores. *Behavior Genetics*, *51*(3), 264–278. https://doi.org/10.1007/s10519-020-10032-w

Chang, C. C., Chow, C. C., Tellier, L. C., Vattikuti, S., Purcell, S. M., & Lee, J. J. (2015). Second-generation PLINK: rising to the challenge of larger and richer datasets. *GigaScience*, *4*(1), 7. https://doi.org/10.1186/s13742-015-0047-8

Delaneau, O., Zagury, J.-F., & Marchini, J. (2013). Improved whole-chromosome phasing for disease and population genetic studies. *Nature Methods*, *10*(1), 5–6. https://doi.org/10.1038/nmeth.2307

Demontis, D., Walters, R. K., Martin, J., Mattheisen, M., Early Lifecourse & Genetic Epidemiology (EAGLE) Consortium, 23andMe Research Team, Als, T. D., Agerbo, E., Baldursson, G., Belliveau, R., Bybjerg-Grauholm, J., Bækvad-Hansen, M., Cerrato, F., Chambert, K., Churchhouse, C., Dumont, A., Eriksson, N., ADHD Working Group of the Psychiatric Genomics Consortium (PGC), Gandal, M., … Neale, B. M. (2019). Discovery of the first genome-wide significant risk loci for attention deficit/hyperactivity disorder. *Nature Genetics*, *51*(1), 63–75. https://doi.org/10.1038/s41588-018-0269-7

Ge, T., Chen, C.-Y., Ni, Y., Feng, Y.-C. A., & Smoller, J. W. (2019). Polygenic prediction via Bayesian regression and continuous shrinkage priors. *Nature Communications*, *10*(1), 1776. https://doi.org/10.1038/s41467-019-09718-5

Howie, B. N., Donnelly, P., & Marchini, J. (2009). A Flexible and Accurate Genotype Imputation Method for the Next Generation of Genome-Wide Association Studies. *PLoS Genetics*, *5*(6), e1000529. https://doi.org/10.1371/journal.pgen.1000529

Loehlin, J. C. (2004). *Latent Variable Models: An Introduction to Factor, Path, and Structural Equation Analysis*. Psychology Press.

Manichaikul, A., Mychaleckyj, J. C., Rich, S. S., Daly, K., Sale, M., & Chen, W.-M. (2010). Robust relationship inference in genome-wide association studies. *Bioinformatics*, *26*(22), 2867–2873. https://doi.org/10.1093/bioinformatics/btq559

McArdle, J. J., & McDonald, R. P. (1984). Some algebraic properties of the Reticular Action Model for moment structures. *British Journal of Mathematical and Statistical Psychology*, *37*(2), 234–251. https://doi.org/10.1111/j.2044-8317.1984.tb00802.x

Okbay, A., Wu, Y., Wang, N., Jayashankar, H., Bennett, M., Nehzati, S. M., Sidorenko, J., Kweon, H., Goldman, G., Gjorgjieva, T., Jiang, Y., Hicks, B., Tian, C., Hinds, D. A., Ahlskog, R., Magnusson, P. K. E., Oskarsson, S., Hayward, C., Campbell, A., … Young, A. I. (2022). Polygenic prediction of educational attainment within and between families from genome-wide association analyses in 3 million individuals. *Nature Genetics*, *54*(4), 437–449. https://doi.org/10.1038/s41588-022-01016-z

Price, A. L., Patterson, N. J., Plenge, R. M., Weinblatt, M. E., Shadick, N. A., & Reich, D. (2006). Principal components analysis corrects for stratification in genome-wide association studies. *Nature Genetics*, *38*(8), 904–909. https://doi.org/10.1038/ng1847

Table S1. The summary of available genotype data in QNTS families

	Two parents	One parent	No parents	All
**MZ families**				
All*	73	19	77	169
**DZ families**				
Two twins	78	41	90	209
One twin	8	7	22	37
All	86	48	112	246
**All families**				
Two twins	151	60	167	378
One twin	8	7	22	37
All	159	67	189	415

* one twin was genotyped in MZ families, the genotypes of MZ twins in the same family were assumed identical

Table S2. Sociodemographic characteristics of the sample, comparison of participants that were included and excluded from the study

	Included families	Excluded families	
**Family sociodemographic characteristics**			
	N = 416	N = 317	
Mother's education, M (SD), years	12.61 (2.88)	12.17 (3.07)	t(552) = 1.823, p = 0.069
Father's education, M (SD), years	12.33 (2.80)	12.33 (3.01)	t(476) = 0.008, p = 0.994
Family income			$\chi^2(1) = 13.513$, p < 0.001
<$50,000	49.0%	64.3%	
≥$50,000	51.0%	35.7%	
Family status			$\chi^2(1) = 1.162$, p = 0.281
two bio parents	83.5%	79.8%	
other	16.5%	20.2%	
Parental language			$\chi^2(2) = 48.321$, p < 0.001
Both Francophone	90.0%	69.8%	
One Francophone	8.9%	19.8%	
Non-Francophone	1.1%	10.5%	
Self-reported parental ethnicity			$\chi^2(1) = 22.353$, p < 0.001
Both White	95.9%	84.0%	
One White	4.1%	16.0%	
**Twins' ADHD symptoms**			
	N = 830	N = 632	
Hyperactivity in early childhood	0.84 (0.40)	0.81 (0.42)	t(895) = 1.203, p = 0.229
Inattention in early childhood	0.63 (0.35)	0.56 (0.37)	t(911) = 3.621, p < 0.001
Hyperactivity in primary school	0.47 (0.46)	0.47 (0.48)	t(666) = -0.189, p = 0.850
Inattention in primary school	0.78 (0.55)	0.88 (0.62)	t(631) = -2.388, p = 0.017

Table S3. Correlations between family members' PGS

	r	95% CI	*Z*	*p*
**ADHD-PGS**				
Mother - father	0.073	[-0.039; 0.183]	1.282	0.200
Child - mother	0.538	[0.464; 0.605]*	11.874*	0.289*
Child - father	0.524	[0.444; 0.595]*	10.909*	0.546*
**EA-PGS**				
Mother - father	0.077	[-0.034; 0.187]	1.354	0.175
Child - mother	0.554	[0.481; 0.619]*	12.211*	0.140*
Child - father	0.585	[-0.512; 0.650]*	12.580*	0.023*

Note. * = Null hypothesis: $r = 0.5$

Table S4. Fit statistics of transmission model

	ep	*df*	-2LL	CFI	TLI	RMSEA	ΔLL	Δ*df*	*p*
**Early childhood**									
ADHD PGS, Hyperactivity	10	1723	1588.47	0.963	0.975	0.029	49.741	37	0.079
ADHD PGS, Inattention	10	1723	1739.02	1.000	1.000	0.000	33.158	37	0.650
EA PGS, Hyperactivity	10	1723	78.30	0.967	0.977	0.029	49.938	37	0.076
EA PGS, Inattention	10	1723	230.88	0.964	0.976	0.029	50.306	37	0.071
**Primary school**									
ADHD PGS, Hyperactivity	10	1721	2128.50	1.000	1.000	0.000	24.580	37	0.941
ADHD PGS, Inattention	10	1721	2172.28	1.000	1.000	0.000	30.026	37	0.785
EA PGS, Hyperactivity	10	1721	622.19	0.988	0.992	0.019	42.556	37	0.244
EA PGS, Inattention	10	1721	645.91	0.989	0.992	0.018	41.800	37	0.270

ep = # of estimated parameters, *df* = degrees of freedom, -2LL = -2 * log-likelihood, CFI = comparative fit index, TLI = Tucker-Lewis index, RMSEA = root mean square error of approximation, ΔLL = difference in log-likelihood between transmission and saturated models, Δ*df* = the difference in degrees of freedom, *p* = *p*-value of chi-squared test

Table S5. Estimates of direct polygenic score effects and variance explained by **ADHD-PGS with 99.2% confidence interval**

	**Hyperactivity**			**Inattention**		
	est.		99.2% CI	est.		99.2% CI
**Early childhood**						
**Direct contributions**						
Child's ADHD-PGS, g'_{tw}	0.016		[-0.125; 0.151]	0.002		[-0.137; 0.140]
Mother's ADHD-PGS, g'_{m}	0.056		[-0.096; 0.195]	0.000		[-0.145; 0.147]
Father's ADHD-PGS, g'_{f}	-0.038		[-0.211; 0.130]	-0.055		[-0.227; 0.112]
**Explained variance**						
Total (%)	0.5		[0.0; 2.6]	0.3		[0.0; 1.7]
Genetic transmission (%)	0.0		[0.0; 0.7]	0.0		[0.0; 0.0]
Genetic nurture (%)	0.5		[-1.1; 3.9]	0.3		[-1.1; 3.1]
**Primary school**						
**Direct contributions**						
Child's ADHD-PGS, g'_{tw}	**0.157**	**	[0.019; 0.284]	0.120	*	[-0.012; 0.247]
Mother's ADHD-PGS, g'_{m}	-0.089		[-0.227; 0.043]	-0.026		[-0.163; 0.107]
Father's ADHD-PGS, g'_{f}	-0.015		[-0.146; 0.135]	-0.009		[-0.146; 0.140]
**Explained variance**						
Total (%)	**1.6**	**	[0.1; 4.3]	1.1	*	[0.0; 3.1]
Genetic transmission (%)	2.5	*	[0.0; 8.0]	1.4	*	[0.0; 6.1]
Genetic nurture (%)	-0.8		[-4.2; 2.1]	-0.3		[-3.4; 1.9]

*Note. est. = estimate; * = p < 0.05, ** = p < 0.008; statistically significant values (p<0.008) are in bold.*

Table S6. Estimates of direct polygenic score effects and variance explained by **EA-PGS with 99.2% confidence interval**

	Hyperactivity			Inattention		
	est.		99.2% CI	est.		99.2% CI
**Early childhood**						
**Direct contributions**						
Child's EA-PGS, g'_{tw}	-0.129	*	[-0.258; 0.009]	**-0.150**	**	**[-0.288; -0.016]**
Mother's EA-PGS, g'_{m}	-0.001		[-0.135; 0.124]	0.013		[-0.132; 0.150]
Father's EA-PGS, g'_{f}	0.004		[-0.141; 0.148]	0.100	*	[-0.034; 0.250]
**Explained variance**						
Total (%)	**1.6**	**	**[0.1; 3.8]**	**1.4**	**	**[0.1; 4.2]**
Genetic transmission (%)	1.7	*	[0.0; 6.7]	2.2	*	[0.0; 8.3]
Genetic nurture (%)	0.0		[-3.6; 3.0]	-0.8		[-4.9; 2.2]
**Primary school**						
**Direct contributions**						
Child's EA-PGS, g'_{tw}	-0.100	*	[-0.231; 0.031]	**-0.153**	**	**[-0.281; -0.014]**
Mother's EA-PGS, g'_{m}	0.020		[-0.118; 0.156]	-0.070		[-0.201; 0.064]
Father's EA-PGS, g'_{f}	-0.091		[-0.226; 0.056]	-0.060		[-0.217; 0.104]
**Explained variance**						
Total (%)	**2.6**	**	**[0.4; 6.1]**	**5.5**	**	**[2.2; 9.7]**
Genetic transmission (%)	1.0		[0.0; 5.3]	2.3	*	[0.0; 7.9]
Genetic nurture (%)	1.6		[-2.3; 6.1]	3.2		[-2.8; 9.1]

*Note. est. = estimate; * = p < 0.05, ** = p < 0.008; statistically significant values (p<0.008) are in bold.*
